# Ibrutinib Resistance Is Reduced by an Inhibitor of Fatty Acid Oxidation in Primary CLL Lymphocytes

**DOI:** 10.3389/fonc.2018.00411

**Published:** 2018-09-26

**Authors:** Gabriela Galicia-Vázquez, Raquel Aloyz

**Affiliations:** ^1^Segal Cancer Center, Lady Davis Institute for Medical Research, Jewish General Hospital, Montreal, QC, Canada; ^2^Division of Experimental Medicine, McGill University, Montreal, QC, Canada; ^3^Department of Oncology, McGill University, Montreal, QC, Canada

**Keywords:** CLL, metabolism, ibrutinib, fatty acid oxidation, drug resistance, del17p

## Abstract

Chronic Lymphocytic Leukemia (CLL) is an incurable disease, characterized by the accumulation of malignant B-lymphocytes in the blood stream (quiescent state) and homing tissues (where they can proliferate). In CLL, the targeting of B-cell receptor signaling through a Burton's tyrosine kinase inhibitor (ibrutinib) has rendered outstanding clinical results. However, complete remission is not guaranteed due to drug resistance or relapse, revealing the need for novel approaches for CLL treatment. The characterization of metabolic rewiring in proliferative cancer cells is already being applied for diagnostic and therapeutic purposes, but our knowledge of quiescent cell metabolism—relevant for CLL cells—is still fragmentary. Recently, we reported that glutamine metabolism in primary CLL cells bearing the del11q deletion is different from their del11q negative counterparts, making del11q cells especially sensitive to glutaminase and glycolysis inhibitors. In this work, we used our primary CLL lymphocyte bank and compounds interfering with central carbon metabolism to define metabolic traits associated with ibrutinib resistance. We observe a differential basal metabolite uptake linked to ibrutinib resistance, favoring glutamine uptake and catabolism. Upon ibrutinib treatment, the redox balance in ibrutinib resistant cells is shifted toward NADPH accumulation, without an increase in glutamine uptake, suggesting alternative metabolic rewiring such as the activation of fatty acid oxidation. In accordance to this idea, the curtailing of fatty acid oxidation by CPT1 inhibition (etomoxir) re-sensitized resistant cells to ibrutinib. Our results suggest that fatty acid oxidation could be explored as a target to overcome ibrutinib resistance.

## Introduction

Chronic Lymphocytic Leukemia (CLL) is the most common adult leukemia in the western world. CLL is characterized by the clonal expansion and accumulation of malignant B-cells in the blood stream and homing tissues (such as bone marrow and lymphoid organs). In the CLL context, positive status of ZAP70, unmutated IgVH, as well as the presence of the chromosomal aberrations *del17p* and *del11q*—spanning TP53 and ATM locus, respectively—are considered as poor outcome predictors (1–3).

One important target for CLL treatment is B-cell receptor (BCR) signaling, since it is involved in B-cell proliferation, differentiation, and migration (4, 5). In CLL, both ligand independent and antigen-induced BCR activation are present, supported by constitutive phosphorylation of BCR signaling components (6, 7). Ibrutinib targets BCR signaling through the irreversible inhibition of the Bruton's tyrosine kinase (BTK) (8). Ibrutinib is among the leading agents used in CLL treatment; however, resistant cases have already arisen, and discontinuation of treatment often triggers disease progression (9, 10). Most relapses to ibrutinib treatment occur due to acquired mutations in BTK or its target PLCγ2, perhaps facilitating the success with alternative targeted therapies (11). In contrast the mechanisms of *DeNovo* resistance to ibrutinib are not well defined.

Therefore, there is a need for additional therapeutic approaches to target CLL.

Aberrant metabolism with respect to normal cells is a hallmark of cancer. Changes in glucose use by cancer cells was the first metabolic alteration described by Warburg, who proposed enhanced use of aerobic glycolysis in cancer cells. Also, altered glutamine use has been reported in cancer (12). More recently atypical use of fatty acids has been described in several types of cancers including glioblastoma, breast cancer, and CLL (13–17). Recently, the characterization of metabolic reprogramming has led to novel therapeutic strategies against a variety of cancers (18). The inhibition of glucose uptake and mitochondrial respiration, using ritonavir and metformin respectively, has been approved for the treatment of Multiple myeloma (19). As well, CB-839—a glutaminase inhibitor—is in clinical trials for AML and ALL treatment (clinicaltrials.gov). In CLL, the use of metformin alone or in combination with ritonavir is being tested at clinical trials (clinicaltrials.gov) (20).

Normal and cancer cells can use glucose and other metabolites, such as fatty acids or amino acids (i.e., glutamine), to obtain energy and building blocks to fulfill their metabolic needs. Thus, metabolite availability has an impact on intracellular processes. In line with this, we have observed differential metabolic responses when cells are exposed to high or limited glucose levels (21). As well, extracellular cystine can modulate intracellular glutamate dependency—through the activation of the glutamate-cystine antiporter: xCT (22). Furthermore, metabolites participate in the regulation of intracellular signaling cascades and redox state amongst others. Alternative metabolic reprogramming has been observed in cancer cell lines exposed to physiological concentrations of metabolites in the media (23).

Although some general assumptions have been reached for cancer cell metabolism; the metabolite utilization in the cell can be quite different depending on the cancel cell type. For instance, transaminases and glutamate dehydrogenase compete to perform glutamate catabolism in breast tumors, depending on the quiescent or proliferative state of the cells (24).

Our knowledge of CLL cell metabolism is still partial, limiting the design of metabolic approaches toward this disease. CLL lymphocytes have an operative glycolytic pathway (21), but they do not follow the Warburg effect. Instead, some reports suggest the preferential use of fatty acid metabolism in these cells (25). In contrast, the increase in glucose metabolism and the use of amino acids on anaplerotic reactions of the TCA cycle has also been proposed (26). The overexpression of oxoglutarate dehydrogenase (OGDC) and isocitrate dehydrogenase (IDH), two enzymes that facilitate the use of the TCA cycle in the forward and reverse directions (i.e., oxidation or reduction of α-ketoglutarate, respectively) (27), support the idea of metabolic plasticity in CLL. In addition, mitochondrial number, mass, activity, and ROS production are increased in CLL compared to normal B-cell lymphocytes (28). Regarding fatty acid metabolism, the production of citrate for fatty acid synthesis is favored via isocitrate dehydrogenase and mitochondrial transhydrogenase (NNT), overexpressed by primary CLL lymphocytes. Interestingly, CLL lymphocytes overexpress enzymes associated with enhanced fatty acid oxidation (FAO) as well (26, 29)

Apart from their known role in DNA damage response and cell cycle control, ATM and TP53 are regulators of central carbon metabolism and redox homeostasis (30–33). Therefore, it is possible that del11q and del17p promote specific metabolic alterations in CLL cells. In line with this, exacerbated mitochondrial respiration in del17p CLL cells has been documented (34). Moreover, we recently reported that glutamine metabolism is altered in del11q positive compared to del11q negative CLL cells. As a result, del11q positive CLL lymphocytes display an increased sensitivity to glutaminase inhibition (35). Metabolic alterations due to cryopreservation have been reported in primary non-malignant human cells isolated from peripheral blood (36–38). In spite of possible limitation, we used cryopreserved primary CLL available in our bank to undertake the task of shedding light into the role of BTK on intrinsic metabolic rewiring in primary CLL lymphocytes and to seek for metabolic traits that can be exploited to reduce *DeNovo* ibrutinib resistance. Noteworthy, available metabolic studies in CLL were carried out using cryopreserved CLL lymphocytes.

## Materials and methods

### Patient samples

Peripheral blood samples donated from 30 patients with a diagnosis of B-CLL attending the Jewish General Hospital Hematology Clinic were utilized in this study. CLL lymphocytes were isolated by Ficoll-Hypaque as previously described (39). This study was carried out in accordance with the recommendations of the published guidelines of the TCPS2—Tri-Council Policy Statement: Ethical Conduct for Research Involving Humans (2014), Jewish General Hospital Research Ethics Committee. The protocol was approved by the Jewish General Hospital Research Ethics Committee. All subjects gave written informed consent in accordance with the Declaration of Helsinki. The characteristics of these samples are summarized on Table 1.

**Table 1:** Patient sample characteristics: ibrutinib sensitivity, IgVH mutation status, diabetic status, Clinical Status, RAI stage, del13q14 status, and del17p status.

**Patient #**	**Ibrutinib sensitivity**	**IgVH status**	**Diabetic status**	**Clinical status**	**RAI stage**	**Del13q status**	**Del17p status**
1	Sen	M	N	T	III	+	–
2	Sen	M	N	NA	IV	NA	–
3	Sen	M	D	T	O	+	–
4	Sen	M	H	T	IV	+	–
5	Sen	M	NA	T	NA	NA	–
6	Sen	U	N	NT	I	+	–
7	Sen	U	N	NT	II	+	–
8	Sen	U	N	NT	II	–	–
9	Sen	U	D	NA	NA	+	–
10	Sen	U	NA	NT	II	+	–
11	Sen	U	NA	NT	NA	–	–
12	Res	M	N	T	II	–	–
13	Res	M	N	T	II	+	–
14	Res	M	N	T	III	–	–
15	Res	M	N	NT	IV	+	–
16	Res	M	N	NA	NA	+	–
17	Res	M	D	NT	III	–	–
18	Res	M	NA	NT	II	–	–
19	Res	M	NA	NT	NA	+	–
20	Res	U	N	NA	I	–	–
21	Res	U	N	NT	IV	–	–
22	Res	U	N	T	IV	–	–
23	Res	U	N	NT	NA	–	–
24	Res	U	D	NA	IV	–	–
25	Res	U	NA	T	II	–	–
26	Res	U	NA	T	NA	–	–
27	Sen	M	NA	NT	II	NA	+
28	Sen	NA	NA	T	NA	+	+
29	Sen	NA	NA	NT	NA	NA	+
30	Res	M	NA	T	I	+	+

*Sen, Sensitive; Res, Resistant; D, Diabetic; H-High glucose; N, Non-diabetic; NA-Not available; M-Mutated; U-Unmutated; T-patients clinically treated; NT-patients not clinically treated; del13q-del13q14. All samples are negative for del11q deletion and naïve for ibrutinib treatment or other tyrosine kinase inhibitors*.

### Cell culture and compound treatments

To date, most of the studies made in CLL were performed with either none or supra-physiological glucose concentrations. In this study, we maintain CLL lymphocytes in physiological (5.5 mM) glucose concentration to describe CLL metabolism. Primary CLL cells were thawed and cultured overnight in AIM-V (GIBCO, 12055-091), 10% FBS at 37°C. Cells were seeded and treated in 5.5 mM glucose RPMI, prepared with a 1:1 mix of RPMI 1640 (Wisent, 350-000) and RPMI 1640 w/o glucose (GIBCO, 11879-020), 10% FBS, 25 mM HEPES, 100 U/mL penicillin/streptomycin, at 37°C and 5% CO_2_. Cells were treated for 24 or 48 h with the following compounds: Ibrutinib (Selleckchem, S2680), Oligomycin A (Sigma, 75351), 2-Deoxy-D-glucose (Sigma, D6134), Ritonavir (Selleckchem, S1185), Compound 968 (Calbiochem, 352010), DHEA (Sigma, D-063), AMPA (Sigma, 324817), Etomoxir (Sigma, E1905), N-Acetyl-L-cysteine (NAC) (Sigma, A7250), Fatty acid supplement (Sigma, F7050). The description of compounds and their targets is shown in Table 2.

**Table 2:** Compounds used to modulate CLL lymphocyte metabolism.

**Name**	**Abbreviation**	**Concentration**	**Target**	**Process Affected**
2-Deoxy-D-glucose (glucose analog)	2DG	2 mM	HK	Glycolysis
Dehydroepiandrosterone	DHEA	10 μM	G6PDH	Pentose phosphate pathway
(Aminomethyl)phosphonic acid	AMPA	10 mM	SHMT1/2	One carbon metabolism
Compound 968	968	10 μM	Glutaminase (GLS)	Glutamine to Glutamate conversion
Oligomycin A	Oligo	2 μM	ATP synthase (Complex V)	Mitochondrial respiration
Ritonavir	Rito	10 μM	GLUT4	Glucose uptake
Etomoxir	Eto	50–100 μM	CPT-1	Fatty acid oxidation
Ibrutinib	Ibru	10 μM	BTK	BCR signaling
N-Acetyl-L-cysteine	NAC	4 mM		Glutathione synthesis and xCT function
Fatty acid supplement	FA			Fatty Acid metabolism

### Viability/mitochondrial reductive capacity assays

Metabolic Activity/Annexin V/Dead Cell Apoptosis Kit with C12 Resazurin, APC annexin V, and SYTOX® Green for Flow Cytometry (Invitrogen, V35114) was used following manufacturer instructions. Briefly, cells were collected 48 h after treatment, spun down at 1300 rpm at 4°C for 5 min. Then, cells were washed in 1X Annexin binding buffer [Invitrogen, V13246 (5X)], spun down at 1300 rpm at 4°C for 5 min, re-suspended in 250 μL of 1X Annexin binding buffer and stained with Sytox green, Resazurin, and APC-Annexin V. Data was collected by flow cytometry (BD FACSAria Fusion cell sorter) at 561-582/15 nm (C12-Resazurin), 488-530/30 nm (Sytox green), 640-670/30 nm (APC-Annexin V). Data analysis was done with FlowJo V.10 software (Systat Software Inc.).

### Glucose, glutamine, glutamate, and ammonia quantification

After 24 h treatment, cell media was collected for metabolite quantification in a NOVA Bioprofile Analyzer 400 at GCRC Metabolomics Core (McGill University). Glucose, glutamine, glutamate, and ammonia uptake (or secretion) were estimated by subtracting their concentration in conditioned media from their concentrations in fresh media and normalized to viable cell number (μM/10^∧^6 cells). Within the concentration of the metabolites above, the pH of conditioned media was documented for each sample. The pH was not affected by any treatment in any of the tested samples.

### ROS levels determination

After 24 h treatment, CellROX® Green Flow Cytometry Assay Kit (Invitrogen, C10492) was used as directed by the manufacturer. Data was collected by flow cytometry (BD FACSAria Fusion cell sorter) at 488–530/30 nm (CellRox), and 640–670/30 nm (Sytox red). Data analysis was done with FlowJo V.10 software (Systat Software Inc.).

### Glutathione concentration determination

Oxidized and Reduced glutathione was determined with GSH/GSSG-Glo Assay (Promega, V6611). Briefly, 1.5 × 10^6^ viable cells were treated for 24 h with selected compounds. After treatment, cells were collected, re-suspended in 125 μL of 1X HBSS (Wisent, 311-512-CL), and 40 μL of each sample was treated to determine oxidized and reduced glutathione according to manufacturer instructions. Measurements were obtained after 1 h, using a FluoStar Optima Reader. A glutathione standard curve was done to calculate glutathione concentration in the samples.

### NAD(P)H/NAD(P) determination

Oxidized and Reduced NAD(P) ratio was determined with NAD(P)/NAD(P)H-Glo Assay (Promega, G9081 [NADPH/NADP] G9071[NADH/NAD]). Briefly, 1.5 × 10^6^ viable cells were treated for 24 h with selected compounds. After treatment, cells were collected, re-suspended in 40 μL of 1X PBS, and treated to determine NAD(P) and NAD(P)H individually according to manufacturer instructions. Measurements were obtained after 1 h, using a FluoStar Optima Reader. The luminescence signal is proportional to the NAD(P) and NAD(P)H amount in the sample.

### Immunoblotting analysis

Cell pellets were collected 24 h after treatment, protein samples were fractionated on 4–12% Criterion™ XT Bis-Tris gels (Bio-Rad, 3450124) in 1X-XT MOPS buffer (BioRad, 161-0788), and transferred to 0.22 μm nitrocellulose membranes (Bio-Rad, 1620112). Antibodies used in this study were directed against: GLUT1 (Fisher, PA5-16793), GLUT4 [IF8] (Santa Cruz, sc-53566), GS (BD Biosciences, 610517), GLS1 [EP7212] (Abcam, ab156876)—to assess for GAC and KGA isoforms, β-Actin (Santa Cruz, sc-1616). Western Blot band quantification was performed using ImageJ 1.49v software (NIH, USA).

### Data analysis

*T*-test and paired *t*-test analysis were performed using Sigma Plot version 13.0. Differences were considered significant if the *p* value was < 0.05.

## Results and discussion

### Ibrutinib sensitive and resistant CLL lymphocytes display differential basal metabolism

**Figure 1:**
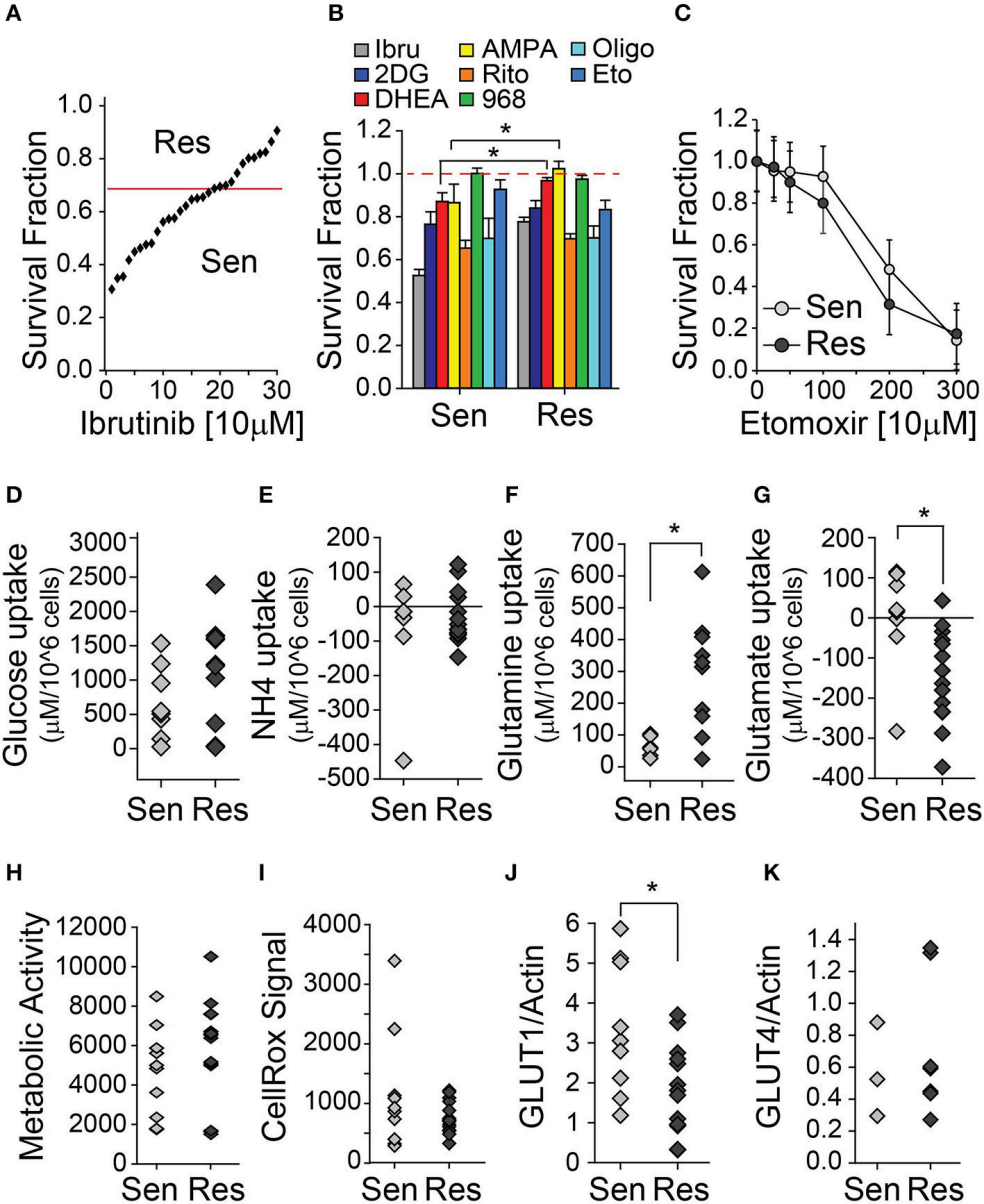
CLL lymphocytes display metabolic differences associated with ibrutinib sensitivity. **(A–C)** Survival fraction (relative to non-treated control [NT]) of CLL lymphocytes treated with metabolic inhibitors for 48 h, **(A)** (*n* = 26), **(B)** (*n* = 15–26), **(C)** (*n* = 11), (mean ± SEM). Basal metabolite uptake differences between ibrutinib sensitive and resistant CLL lymphocytes, **(D)** Glucose (*n* = 20), **(E)** Ammonia (*n* = 22), **(F)** Glutamine (*n* = 16), **(G)** Glutamate (*n* = 23). Negative values indicate metabolite excretion to the media. **(H)** Basal raw (Resazurin) signal of mitochondrial reductive capacity of ibrutinib sensitive and resistant CLL lymphocytes (*n* = 25). **(I)** Basal raw ROS (CellRox) signal of ibrutinib sensitive and resistant CLL lymphocytes (*n* = 25). Basal protein expression of ibrutinib sensitive and resistant CLL lymphocytes (relative to actin signal) **(J)** GLUT1 (*n* = 22) and **(K)** GLUT4 (*n* = 10), *p < 0.05.

To study the metabolic traits associated with ibrutinib resistance, we used 30 samples from our primary CLL lymphocyte bank (Table 1). All CLL samples were del11q negative, naïve to ibrutinib treatment, and 4 were del17p positive. Forty-eight hours of *in vitro* treatment with 10 μM ibrutinib resulted in a heterogeneous decrease in the survival fraction among the samples tested. We established ibrutinib sensitive and resistant sample subsets using the median of the sample population (0.68) as a cut-off (Figure 1A). An initial assessment of the effects of metabolic inhibition was done by treating del17p negative CLL lymphocytes with a series of inhibitors (Table 2) for 48 h. Ibrutinib sensitive cells were slightly sensitive to both pentose phosphate pathway and one carbon cycle inhibition compared to ibrutinib resistant cells, suggesting that the former subset is more dependent on glucose metabolism for survival (Figure 1B). In contrast, although not statistically significant, cell viability in ibrutinib resistant lymphocytes tended to be more affected by the inhibition of fatty acid oxidation pathway—using etomoxir (Figure 1C).

Ibrutinib sensitive and resistant CLL lymphocytes had comparable levels of basal glucose uptake and extracellular accumulation of ammonia (Figures 1D,E). However, ibrutinib resistant cells had a higher basal glutamine uptake (Figure 1F), accompanied by increased basal glutamate secretion when compared to ibrutinib sensitive cells (Figure 1G). The latter suggests heightened intracellular amino acid catabolism in ibrutinib sensitive cells, using transamination to obtain glutamate, followed by ammonia and α-ketoglutarate production by glutamate dehydrogenase (GDH). The expression of enzymes involved in glutamine metabolism—such as glutamate dehydrogenase, glutaminase (GLS) or glutamine synthetase (GS)—was comparable in CLL lymphocytes when analyzed vis a vis ibrutinib sensitivity (*unpublished results*). As well, mitochondrial metabolic activity and ROS generation were similar in both subsets, suggesting distinct compensation mechanisms to maintain mitochondrial homeostasis (Figures 1H,I). Additionally, basal glucose transporter (GLUT) 1 expression is higher in ibrutinib sensitive cells (Figure 1J), which could contribute to an increased dependence on glucose metabolism compared to their resistant counterparts. In line with this is the increased sensitivity of ibrutinib sensitive cells to pentose phosphate and one carbon cycle pathways. In contrast, GLUT4 expression was similar in both subsets, which is in line with a comparable cytotoxic effect of ritonavir (GLUT4 inhibitor) on CLL lymphocytes, regardless of ibrutinib sensitivity (Figure 1K). A differential expression, subcellular localization, or activity of glucose transporters might account for the equivalent levels of basal glucose uptake observed in ibrutinib sensitive and resistant subsets.

### Ibrutinib sensitive and resistant CLL lymphocytes metabolic response to BTK inhibition

**Figure 2:**
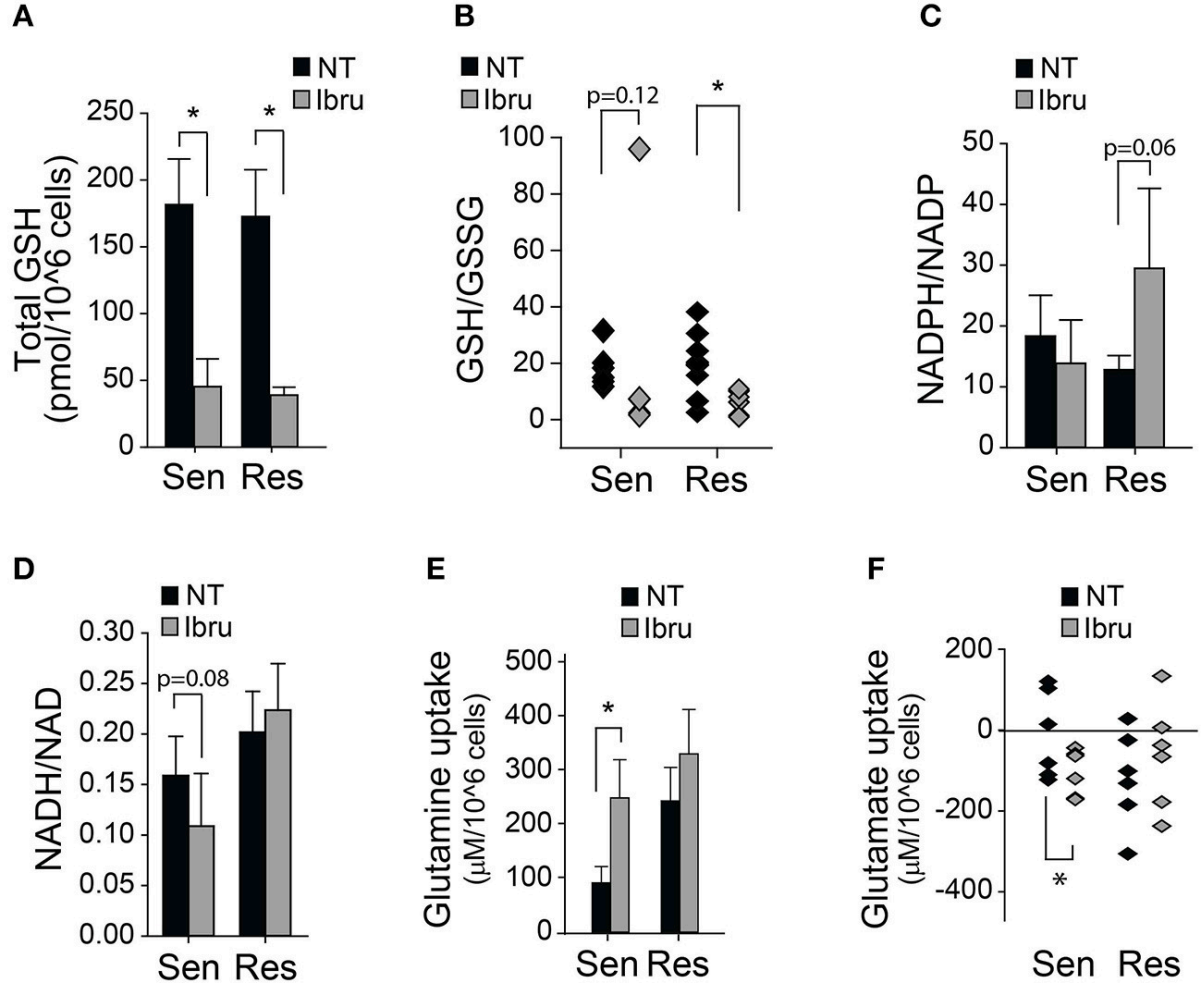
Metabolic and Redox effects of ibrutinib on CLL lymphocytes. **(A)** Quantification of total glutathione after 24 h treatment with ibrutinib (*n* = 15), (mean ± SEM). **(B)** Reduced/Oxidized glutathione ratio after 24 h treatment with ibrutinib (*n* = 14). **(C)** NADPH/NADP ratio after 24 h of ibrutinib treatment (*n* = 8) (mean ± SEM). **(D)** NADH/NAD ratio after 24 h of ibrutinib treatment (*n* = 11) (mean ± SEM). Metabolite uptake after 24 h of ibrutinib treatment. **(E)** Glutamine (*n* = 21) and **(F)** Glutamate (*n* = 20) (mean ± SEM), *p < 0.05.

Recently, we reported that ibrutinib increases glucose as well as glutamine uptake; and that the supplementation with a cysteine analog (NAC) could rescue ibrutinib-induced cytotoxicity and ibrutinib-induced ROS levels (35). To further characterize the response of primary CLL lymphocytes to ibrutinib treatment we evaluated the redox status of glutathione (GSH), NAD, and NADP. The decrease in total glutathione levels upon ibrutinib treatment was comparable in both subsets (Figure 2A), as well as the decrease in reduced glutathione levels (Figure 2B), suggesting that the rise in ROS levels upon ibrutinib treatment might result from reduced glutathione synthesis rather than diminished NADPH availability for glutathione regeneration. In fact, ibrutinib did not affect the NADPH/NADP ratio in sensitive samples and tended to increase it in resistant samples (Figure 2C). In contrast, NADH/NAD ratio was not affected by ibrutinib in resistant samples but tended to decrease in ibrutinib sensitive samples (Figure 2D). The latter suggests that ibrutinib reduces the bio-energetic capacity in sensitive cells, which might partially contribute to the observed cell death in this subset after 48 h treatment.

Ibrutinib sensitive cells increased their glutamine uptake in response to ibrutinib (Figure 2E). This effect was accompanied by heightened glutamate secretion, possibly due to increased glutamine deamination (Figure 2F). In contrast, glutamine uptake was not affected in response to ibrutinib in the resistant subset (Figure 2E), but extracellular glutamate accumulation was reduced in 4 out of 6 analyzed samples (Figure 2F). The latter suggests that ibrutinib favors glutamate depletion in resistant cells, perhaps by increasing glutamate dehydrogenase activity, promoting NADPH production (Figure 2C), and redirecting α-ketoglutarate (α-KG) to feed the TCA cycle. Since NADPH is not likely consumed to regenerate glutathione levels in resistant samples after ibrutinib treatment, its use in different pathways could be proposed.

### Role of fatty acid oxidation on CLL lymphocyte ibrutinib resistance

The contribution of fatty acid metabolism to CLL metabolic plasticity has been previously explored. Fatty acid synthesis is favored via citrate production by isocitrate dehydrogenase, in addition to the over-expression of enzymes associated with enhanced fatty acid oxidation (26, 29). Recently, ibrutinib-induced inhibition on free fatty acid synthesis has been reported (40), supporting the idea that ibrutinib plays a role in CLL cell metabolism.

**Figure 3:**
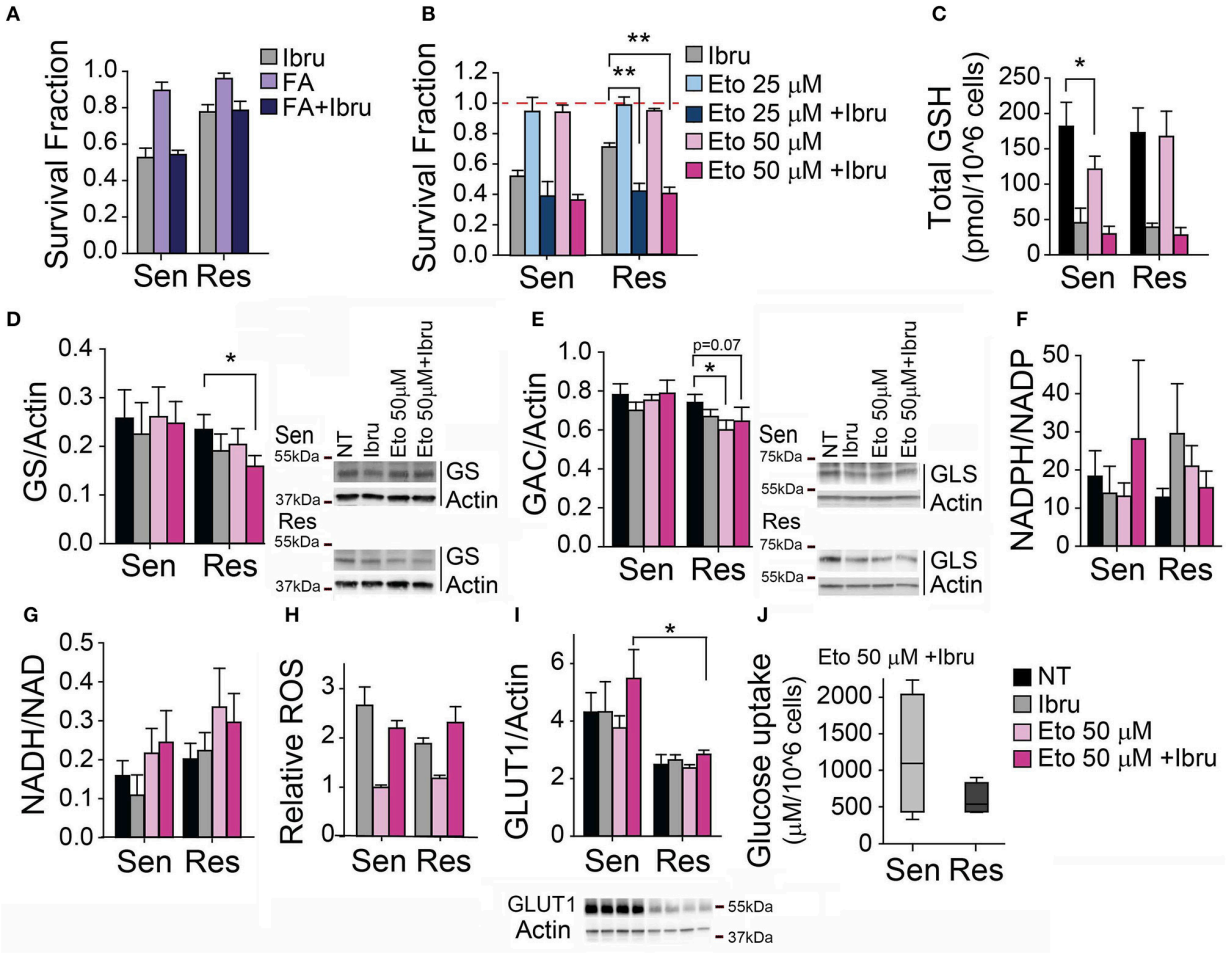
Inhibition of FAO decreases ibrutinib-induced cytotoxicity in CLL primary lymphocytes. **(A)** Survival fraction (relative to non-treated control) after 48 h treatment with ibrutinib alone or in combination with Fatty acids (FA), (*n* = 10). **(B)** Survival fraction (relative to non-treated control) after 48 h treatment with etomoxir alone or in combination with ibrutinib, (*n* = 12). **(C)** Total glutathione levels after 24 h treatment with etomoxir alone or in combination with ibrutinib (*n* = 11). Protein expression levels (relative to actin) after 24 h treatment with etomoxir alone or in combination with ibrutinib **(D)** GS (*n* = 11), **(E)** GAC (*n* = 10), **(I)** GLUT1 (*n*=10). **(F)** NADPH/NADP ratio (*n* = 8), and **(G)** NADH/NAD ratio (*n* = 11) after 24 h treatment with etomoxir alone or in combination with ibrutinib. **(H)** Relative ROS levels after 48 h treatment with etomoxir alone or in combination with ibrutinib (*n* = 12), (mean ± SEM). **(J)** Glucose uptake after 24 h treatment with ibrutinib and etomoxir (*n* = 8). *p < 0.05, **p < 0.001.

Given that a compensatory activation of fatty acid oxidation may also contribute to sustain TCA cycle and to increase NADPH intracellular pools, we propose that resistant cells could increase fatty acid oxidation flux as an alternative to elevated glutamine uptake in the cell after ibrutinib treatment. It is unlikely that ibrutinib effects are attenuated by fatty acid uptake, since Fatty acid supplementation to cells treated with ibrutinib does not rescue cell viability in any subset (Figure 3A). Metabolic rewiring involving glucose-derived carbons could also be considered. For instance, the increase in acetyl-CoA availability could be promoted via Pyruvate dehydrogenase, and/or by redirecting glyceraldehyde-3-phosphate carbons for triglyceride production—via Triosephosphate isomerase (TPI), overexpressed in CLL (27).

To assess the association of ibrutinib resistance with fatty acid metabolism rewiring, we evaluated the combination of ibrutinib and etomoxir. Etomoxir inhibits carnitine palmitoyltransferase I (CPT-1), hampering mitochondrial β-oxidation of fatty acids. We utilize etomoxir at concentrations commonly used in preclinical studies involving primary leukemic cells (41–45). We observed that the use of non-cytotoxic concentrations of etomoxir in the presence of 10 μM ibrutinib did not modify ibrutinib-induced cytotoxicity in ibrutinib sensitive subset (Figure 3B). Of note, non-cytotoxic concentrations of etomoxir decrease total GSH by 30% in sensitive cells (Figure 3C), perhaps reflecting the partial redirection of available glutamate to α-ketoglutarate production for TCA cycle homeostasis maintenance (at the expense of cystine import). Remarkably, etomoxir re-sensitized resistant cells to ibrutinib-induced cytotoxicity (Figure 3B). We found that the protein expression of enzymes related to glutamine metabolism is affected when ibrutinib resistant cells were treated simultaneously with ibrutinib and etomoxir. GS expression is decreased by 30% (Figure 3D), while glutaminase isoform GAC slightly decreased (Figure 3E), showing a preference to maintain intracellular glutamate pools over glutamine production. In these conditions, the reduced/oxidized NADP and NAD metabolite ratios remained at basal levels, regardless of ibrutinib sensitivity status (Figures 3F,G). However, heightened ROS levels were observed when treating CLL lymphocytes with ibrutinib plus etomoxir, similar to their ibrutinib-only treated counterparts (Figure 3H), a possible consequence of decreased total glutathione (Figure 3C).

The preservation of NADH/NAD ratio basal level could be a consequence of the utilization of glutamate by ibrutinib resistant cells to feed the TCA cycle, favoring the flux toward the oxidative reactions and providing reducing equivalents for mitochondrial respiration. In addition, these cells could promote PEPCK activity to use oxaloacetate for supplying reagents to one carbon cycle, compensating for decreased FAO-derived NADPH.

Interestingly, upon ibrutinib plus etomoxir treatment, sensitive cells displayed a higher expression of GLUT1 when compared to resistant CLL cells (Figure 3I). This is reflected in a tendency of ibrutinib sensitive cells to uptake more glucose than resistant cells upon ibrutinib plus etomoxir treatment (Figure 3J). The increased influx of glucose-derived carbons to one carbon cycle and the mitochondria might compensate for the lack of fatty acid-derived NADPH and mitochondrial-dependent NADH pools, respectively (Figures 3F,G)—revealing differential metabolic responses between subsets.

Upon simultaneous inhibition of BTK and FAO, the reductive capacity associated to NAD and NADP is maintained, accompanied by a decrease of reduced glutathione in both subsets. Ibrutinib-induced cytotoxicity is associated with increased ROS levels in CLL lymphocytes (35). However, ibrutinib sensitive and resistant cell subsets exhibit similar ROS levels upon ibrutinib treatment (with or without etomoxir), revealing differential control of oxidative stress and/or cell death mechanisms. The evaluation of the expression and/or activity of enzymes involved in oxidative stress response—such as superoxide dismutase, catalase, or glutathione peroxidase—remains to be explored. As well, the level of ROS damage measuring lipid peroxidation could be estimated.

### TP53 metabolic rewiring favors a catabolic metabolism

**Figure 4:**
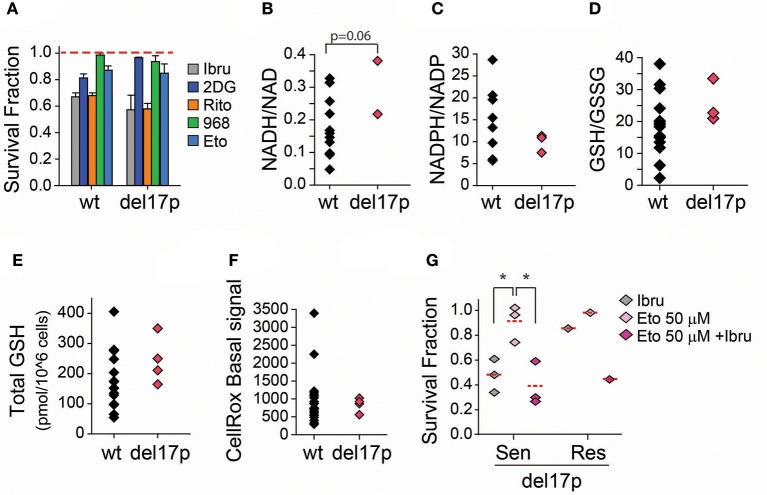
Effect of FAO inhibition in ibrutinib treated CLL lymphocytes positive for del17p. **(A)** Survival fraction (relative to non-treated control [NT]) after 48 h treatment with the indicated metabolic inhibitors (*n* = 20–30) (mean ± SEM). **(B)** Basal NADH/NAD ratio (*n* = 11), and **(C)** Basal NADPH/NADP ratio (*n* = 14) of CLL lymphocytes negative and positive to del17p. **(D)** Reduced/Oxidized glutathione ratio (*n* = 18), and **(E)** Basal total glutathione levels (*n* = 19) of CLL lymphocytes negative and positive to del17p. **(F)** Basal raw ROS (CellRox) signal on CLL lymphocytes negative and positive to del17p (*n* = 29). **(G)** Survival fraction (relative to non-treated control) after 48 h treatment with combinations of ibrutinib and etomoxir (*n* = 4). wt -del17p negative CLL lymphocytes.

TP53 is a modulator of central carbon metabolism and redox homeostasis (30–33). Since mitochondrial respiration and biogenesis are exacerbated in CLL lymphocytes positive for a deletion spanning the TP53 locus (*del17p*) it is likely that TP53 plays an important role in CLL metabolism as well (34). To begin to understand alterations in central carbon metabolism specifically associated with *del17p* in CLL, four samples from our primary cell bank were challenged with metabolic inhibitors (Table 1). Curtailing glycolysis (2DG), glucose uptake (ritonavir), glutaminolysis (Compound 968), or FAO (etomoxir) was cytotoxic to *del17p* samples to the same extent as to *del17p* negative samples (Figure 4A). Conversely, basal differential metabolic reprogramming was revealed by the evaluation of key indicators of the cell redox state. NADH/NAD ratio is preferentially enhanced while the opposite was observed for NADPH/NADP levels in this subset, with respect to *del17p* negative cells (Figures 4B,C). Decreased NADPH/NADP could be attributed to heightened glutathione regeneration. In line with this, we observed that enhanced GSH/GSSG ratio is associated with *del17p*, but not with total glutathione levels (Figures 4D,E). Such metabolic organization promoted by *del17p* favors the maintenance of elevated mitochondrial metabolism, previously reported in a model of TP53 deficient CLL cells (34). In agreement with this, differences in basal ROS production are not associated with *del17p* status (Figure 4F).

Ibrutinib is considered a therapeutic option to treat CLL cases positive for del17p. When we analyzed the samples from our primary lymphocyte databank, three out of the four del17p positive samples were sensitive to ibrutinib (Figure 4G). Although increased number of samples should be considered, we observed that the inhibition of FAO re-sensitized our del17p CLL ibrutinib resistant sample to ibrutinib (Figure 3B), as it was observed for del17p negative cells. Our ongoing work is aimed at investigating the rewiring of glutamate, glucose and fatty acid metabolism in del17p positive cells upon ibrutinib treatment.

## Conclusions and model

**Figure 5:**
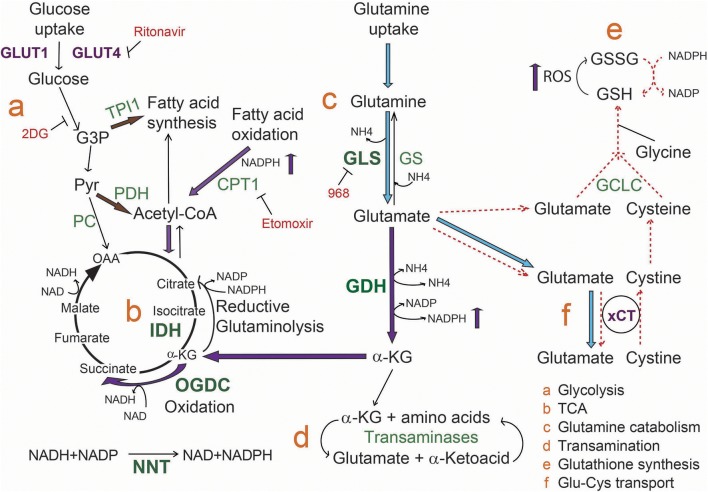
Model for metabolic rewiring associated to ibrutinib resistance in CLL lymphocytes. Upon ibrutinib treatment, ibrutinib resistant cells initiate a compensation mechanism increasing fatty acid oxidation metabolism as well as α-KG production from glutamate, to maintain mitochondrial homeostasis. In line with this, we propose that α-KG follows preferentially an oxidation process in the TCA. Transporters are colored in purple, enzymes in green, and inhibitors in red. Enzymes overexpressed in CLL lymphocytes are in bold green characters. Blue arrows indicate reactions increased in basal ibrutinib resistant cells compared to the sensitive subset. Purple arrows represent the processes that are increased in resistant cells upon ibrutinib treatment. Dashed red arrows represent the processes inhibited upon ibrutinib treatment. Brown arrows indicate the proposed alternative metabolic rewiring to increase fatty acid and Acetyl-CoA availability.

Based on the obtained results, we propose that ibrutinib downregulates cell metabolism at the TCA cycle level, causing a decrease in glutamate availability by increasing its catabolism to feed the TCA cycle. In these conditions, ibrutinib resistant CLL lymphocytes carry out a compensation mechanism involving increased fatty acid oxidation flux. As well, glutamate export is inhibited, likely to sustain intracellular glutamate pools (Figure 5). The re-sensitization of the resistant subset upon simultaneous treatment with etomoxir and ibrutinib, reveals that fatty acid metabolism could be explored as a therapeutic target to overcome ibrutinib resistance.

## Author contributions

GG-V performed the experiments, RA and GG-V did the data analysis and prepared the manuscript.

### Conflict of interest statement

The authors declare that the research was conducted in the absence of any commercial or financial relationships that could be construed as a potential conflict of interest.

## References

[1] Oscier DG Gardiner AC Mould SJ Glide S Davis ZA Ibbotson RE. Multivariate analysis of prognostic factors in CLL: clinical stage, IGVH gene mutational status, and loss or mutation of the p53 gene are independent prognostic factors. Blood (2002) 100:1177–84. 12149195

[2] Amin NA Balasubramanian S Saiya-Cork K Shedden K Hu N Malek SN. Cell-intrinsic determinants of ibrutinib-induced apoptosis in chronic lymphocytic leukemia. Clin Cancer Res. (2017) 23:1049–59. 10.1158/1078-0432.CCR-15-2921 27535981 PMC5315657

[3] Ensafi AA Amini M Rezaei B Talebi M. A novel diagnostic biosensor for distinguishing immunoglobulin mutated and unmutated types of chronic lymphocytic leukemia. Biosens Bioelectron. (2016) 77:409–15. 10.1016/j.bios.2015.09.063 26436328

[4] Hendriks RW Yuvaraj S Kil LP. Targeting Bruton's tyrosine kinase in B cell malignancies. Nat Rev Cancer (2014) 14:219–32. 10.1038/nrc3702 24658273

[5] Burger J. B-cell receptor signaling in chronic lymphocytic leukemia and other B-cell malignancies. Clin Adv Hematol Oncol. (2016) 14:55–65. 27057669

[6] Dühren-von Minden M Übelhart R Schneider D Wossning T Bach MP Buchner M. Chronic lymphocytic leukaemia is driven by antigen-independent cell-autonomous signalling. Nature (2012) 489:309–12. 10.1038/nature11309 22885698

[7] Gobessi S Laurenti L Longo P Carsetti L Berno V Sica S. Inhibition of constitutive and BCR-induced Syk activation downregulates Mcl-1 and induces apoptosis in chronic lymphocytic leukemia B cells. Leukemia (2009) 23:686–97. 10.1038/leu.2008.346 19092849

[8] Ponader S Chen SS Buggy JJ Balakrishnan K Gandhi V Wierda WG. The Bruton tyrosine kinase inhibitor PCI-32765 thwarts chronic lymphocytic leukemia cell survival and tissue homing *in vitro* and *in vivo*. Blood (2012) 119:1182–9. 10.1182/blood-2011-10-386417 22180443 PMC4916557

[9] Young RM Staudt LM. Ibrutinib treatment of CLL: the cancer fights back. Cancer Cell (2014) 26:11–3. 10.1016/j.ccr.2014.06.023 25026208 PMC4199743

[10] Woyach JA Johnson AJ. Targeted therapies in CLL: mechanisms of resistance and strategies for management. Blood (2015) 126:471–7. 10.1182/blood-2015-03-585075 26065659 PMC4513250

[11] Woyach JA. How I manage ibrutinib-refractory chronic lymphocytic leukemia. Blood (2017) 129:1270–4. 10.1182/blood-2016-09-693598 28096090 PMC5345730

[12] Zhang J Pavlova NN Thompson CB. Cancer cell metabolism: the essential role of the nonessential amino acid, glutamine. EMBO J. (2017) 36:1302–15. 10.15252/embj.201696151 28420743 PMC5430235

[13] Abate M Laezza C Pisanti S Torelli G Seneca V Catapano G. Deregulated expression and activity of Farnesyl Diphosphate Synthase (FDPS) in Glioblastoma. Sci Rep. (2017) 7:14123. 10.1038/s41598-017-14495-6 29075041 PMC5658376

[14] Kuo CY Ann DK. When fats commit crimes: fatty acid metabolism, cancer stemness and therapeutic resistance. Cancer Commun. (2018) 38:47. 10.1186/s40880-018-0317-9 29996946 PMC6042406

[15] Li Z Zhang H. Reprogramming of glucose, fatty acid and amino acid metabolism for cancer progression. Cell Mol Life Sci. (2016) 73:377–92. 10.1007/s00018-015-2070-4 26499846 PMC11108301

[16] Monaco ME. Fatty acid metabolism in breast cancer subtypes. Oncotarget (2017) 8:29487–500. 10.18632/oncotarget.15494 28412757 PMC5438746

[17] Bilban M Heintel D Scharl T Woelfel T Auer MM Porpaczy E. Deregulated expression of fat and muscle genes in B-cell chronic lymphocytic leukemia with high lipoprotein lipase expression. Leukemia (2006) 20:1080–8. 10.1038/sj.leu.2404220 16617321

[18] Hanahan D Weinberg RA. Hallmarks of cancer: the next generation. Cell (2011) 144:646–74. 10.1016/j.cell.2011.02.013 21376230

[19] Dalva-Aydemir S Bajpai R Martinez M Adekola KU Kandela I Wei C. Targeting the metabolic plasticity of multiple myeloma with FDA-approved ritonavir and metformin. Clin Cancer Res. (2015) 21:1161–71. 10.1158/1078-0432.CCR-14-1088 25542900 PMC5571862

[20] Adekola KU Dalva Aydemir S Ma S Zhou Z Rosen ST Shanmugam M. Investigating and targeting chronic lymphocytic leukemia metabolism with the human immunodeficiency virus protease inhibitor ritonavir and metformin. Leuk Lymphoma (2015) 56:450–9. 10.3109/10428194.2014.922180 24828872 PMC4868500

[21] Martinez Marignac VL Smith S Toban N Bazile M Aloyz R. Resistance to Dasatinib in primary chronic lymphocytic leukemia lymphocytes involves AMPK-mediated energetic re-programming. Oncotarget (2013) 4:2550–66. 10.18632/oncotarget.1508 24334291 PMC3926848

[22] Muir A Danai LV Gui DY Waingarten CY Lewis CA Vander Heiden MG. Environmental cystine drives glutamine anaplerosis and sensitizes cancer cells to glutaminase inhibition. Elife. (2017) 6:e27713. 10.7554/eLife.27713 28826492 PMC5589418

[23] Cantor JR Abu-Remaileh M Kanarek N Freinkman E Gao X Louissaint A Jr. Physiologic medium rewires cellular metabolism and reveals uric acid as an endogenous inhibitor of UMP synthase. Cell (2017) 169:258–72.e17. 10.1016/j.cell.2017.03.023 28388410 PMC5421364

[24] Coloff JL Murphy JP Braun CR Harris IS Shelton LM Kami K. Differential glutamate metabolism in proliferating and quiescent mammary epithelial cells. Cell Metab. (2016) 23:867–80. 10.1016/j.cmet.2016.03.016 27133130

[25] Rozovski U Hazan-Halevy I Barzilai M Keating MJ Estrov Z. Metabolism pathways in chronic lymphocytic leukemia. Leuk Lymphoma (2016) 57:758–65. 10.3109/10428194.2015.1106533 26643954 PMC4794359

[26] Tili E Michaille JJ Luo Z Volinia S Rassenti LZ Kipps TJ. The down-regulation of miR-125b in chronic lymphocytic leukemias leads to metabolic adaptation of cells to a transformed state. Blood (2012) 120:2631–8. 10.1182/blood-2012-03-415737 22723551 PMC3460685

[27] Zelenetz AD. Chronic lymphocytic leukemia: individualizing treatment approach. J Natl Compr Canc Netw. (2017) 15:713–5. 10.6004/jnccn.2017.0081 28515252

[28] Jitschin R Hofmann AD Bruns H Giessl A Bricks J Berger J. Mitochondrial metabolism contributes to oxidative stress and reveals therapeutic targets in chronic lymphocytic leukemia. Blood (2014) 123:2663–72. 10.1182/blood-2013-10-532200 24553174

[29] Mayer RL Schwarzmeier JD Gerner MC Bileck A Mader JC Meier-Menches SM. Proteomics and metabolomics identify molecular mechanisms of aging potentially predisposing for chronic lymphocytic leukemia. Mol Cell Proteomics (2018) 17:290–303. 10.1074/mcp.RA117.000425 29196338 PMC5795392

[30] Zaugg K Yao Y Reilly PT Kannan K Kiarash R Mason J. Carnitine palmitoyltransferase 1C promotes cell survival and tumor growth under conditions of metabolic stress. Genes Dev. (2011) 25:1041–51. 10.1101/gad.1987211 21576264 PMC3093120

[31] Berkers CR Maddocks OD Cheung EC Mor I Vousden KH. Metabolic regulation by p53 family members. Cell Metabol. (2013) 18:617–33. 10.1016/j.cmet.2013.06.019 23954639 PMC3824073

[32] Krüger A Ralser M. ATM is a redox sensor linking genome stability and carbon metabolism. Sci Signal. (2011) 4:pe17. 10.1126/scisignal.2001959 21467295

[33] Vousden KH Ryan KM. p53 and metabolism. Nat Rev Cancer (2009) 9:691–700. 10.1038/nrc2715 19759539

[34] Ogasawara MA Liu J Pelicano H Hammoudi N Croce CM Keating MJ. Alterations of mitochondrial biogenesis in chronic lymphocytic leukemia cells with loss of p53. Mitochondrion (2016) 31:33–9. 10.1016/j.mito.2016.09.001 27650502 PMC5108679

[35] Galicia-Vazquez G Smith S Aloyz R. Del11q-positive CLL lymphocytes exhibit altered glutamine metabolism and differential response to GLS1 and glucose metabolism inhibition. Blood Cancer J. (2018) 8:13. 10.1038/s41408-017-0039-2 29367649 PMC5802573

[36] Keane KN Calton EK Cruzat VF Soares MJ Newsholme P. The impact of cryopreservation on human peripheral blood leucocyte bioenergetics. Clin Sci. (2015) 128:723–33. 10.1042/CS20140725 25597817

[37] D'alessandro A Gray AD Szczepiorkowski ZM Hansen K Herschel LH Dumont LJ. Red blood cell metabolic responses to refrigerated storage, rejuvenation, and frozen storage. Transfusion (2017) 57:1019–30. 10.1111/trf.14034 28295356

[38] Johnson L Tan S Wood B Davis A Marks DC. Refrigeration and cryopreservation of platelets differentially affect platelet metabolism and function: a comparison with conventional platelet storage conditions. Transfusion (2016) 56:1807–18. 10.1111/trf.13630 27158813

[39] Amrein L Panasci L Gibson SB Johnston JB Soulieres D Aloyz R. Primary del 17 chronic lymphocytic leukaemia lymphocytes are hypersensitive to dasatinib *in vitro*. Br J Haematol. (2009) 147:396–8. 10.1111/j.1365-2141.2009.07814.x 19758122 PMC2774145

[40] Rozovski U Harris DM Li P Liu Z Jain P Ferrajoli A Ibrutinib inhibits free fatty acid metabolism in chronic lymphocytic leukemia. Leuk Lymphoma (2018) 21:1–6. 10.1080/10428194.2018.1439167 PMC6135679 29465264

[41] Estan MC Calvino E Calvo S Guillen-Guio B Boyano-Adanez Mdel C de Blas E. Apoptotic efficacy of etomoxir in human acute myeloid leukemia cells. cooperation with arsenic trioxide and glycolytic inhibitors, and regulation by oxidative stress and protein kinase activities. PLoS ONE (2014) 9:e115250. 10.1371/journal.pone.0115250 25506699 PMC4266683

[42] Samudio I Harmancey R Fiegl M Kantarjian H Konopleva M Korchin B. Pharmacologic inhibition of fatty acid oxidation sensitizes human leukemia cells to apoptosis induction. J Clin Invest. (2010) 120:142–56. 10.1172/JCI38942 20038799 PMC2799198

[43] Ricciardi MR Mirabilii S Allegretti M Licchetta R Calarco A Torrisi MR. Targeting the leukemia cell metabolism by the CPT1a inhibition: functional preclinical effects in leukemias. Blood (2015) 126:1925–9. 10.1182/blood-2014-12-617498 26276667

[44] Holubarsch CJ Rohrbach M Karrasch M Boehm E Polonski L Ponikowski P. A double-blind randomized multicentre clinical trial to evaluate the efficacy and safety of two doses of etomoxir in comparison with placebo in patients with moderate congestive heart failure: the ERGO (etomoxir for the recovery of glucose oxidation) study. Clin Sci. (2007) 113:205–12. 10.1042/CS20060307 17319797

[45] Kerner J Zaluzec E Gage D Bieber LL. Characterization of the malonyl-CoA-sensitive carnitine palmitoyltransferase (CPTo) of a rat heart mitochondrial particle. evidence that the catalytic unit is CPTi. J Biol Chem. (1994) 269:8209–19. 8132545

